# Soil heavy metal contamination assessment in the Hun-Taizi River watershed, China

**DOI:** 10.1038/s41598-020-65809-0

**Published:** 2020-05-26

**Authors:** Wei Zhang, Miao Liu, Chunlin Li

**Affiliations:** 1 College of Land and Environment, Shenyang Agricultural University, Shenyang, 110161 China; 2 CAS Key Laboratory of Forest Ecology and Management, Institute of Applied Ecology, Chinese Academy of Sciences, Shenyang, 110164 China

**Keywords:** Environmental impact, Risk factors

## Abstract

The Hun-Taizi River watershed includes the main part of the Liaoning central urban agglomeration, which contains six cities with an 80-year industrial history. A total of 272 samples were collected from different land use areas within the study area to estimate the concentration levels, spatial distributions and potential sources of arsenic (As), cadmium (Cd), chromium (Cr), copper (Cu), mercury (Hg), nickel (Ni), lead (Pb) and zinc (Zn) with a geographic information system (GIS), principal component analysis (PCA) and canonical correspondence analysis (CCA). Only the concentration of Cd was over the national standard value (GB 15618–2018). However, the heavy metal concentrations at 24.54%, 71.43%, 63.37%, 85.71, 70.33%, 53.11%, and 72.16% of the sampling points were higher than the local soil background values for As, Cd, Cr, Cu, Hg, Ni, Pb and Zn, respectively, which were used as standard values in this study. The maximal values of Cd (16.61 times higher than the background value) and Hg (12.18 times higher than the background value) had high concentrations, while Cd was present in the study area at higher values than in some other basins in China. Cd was the primary pollutant in the study area due to its concentration and potential ecological risk contribution. The results of the potential ecological risk index (RI) calculation showed that the overall heavy metal pollution level of the soil was considerably high. Three groups of heavy metals with similar distributions and sources were identified through PCA. The results of the CCA showed that the distribution of mines was the strongest factor affecting the distributions of Ni, As, Zn, Pb, and Cd. However, Cu was strongly influenced by the distance to the nearest river. These findings can provide scientific support for critical planning and strategies for soil pollution control and removal to support the sustainable development of the study area.

## Introduction

China has undergone rapid urbanization in the last several decades. Over twenty urban agglomerations have formed, and many urban agglomerations include cities with heavy industry^1^. Sustained and intensive human activities, especially agricultural and industrial production, in urban agglomeration areas have brought heavy metal pollution to surface soils in many areas^2,3^. Moreover, most cities within the same urban agglomeration are usually located within one watershed, and soil pollution migrates through hydrologic processes, soil erosion and agricultural irrigation. Soil is a sink for heavy metal pollutants; meanwhile, metal contaminants will transfer to other places through hydrological and soil erosion processes, especially within the same watershed. The heavy metals in urban agglomeration soils may result in excessive increases in human exposure to heavy metals due to food production and general human activities in the area^4^. The assessment of heavy metal distributions in soils and their influencing factors could provide information to repair regional environmental quality and improve ecosystem health^5^.

Studies have been performed to survey and estimate soil heavy metal contamination in many different regions, such as the Concórdia River watershed^6^, the upper Yangtze basin^7^, the Mustafakemalpasa stream basin^8^, the Raohe basin^9^, the Yellow River delta^10^, the Yangtze River delta^11^, the Mississippi River delta^12^, agricultural land^13,14^, urban areas^15^, mining regions^16,17^ and road traffic areas^18^. Ecological risk assessments^19,20^, health risk assessments^21^ and source identification^22^ have been carried out in many studies. Soil heavy metal pollution in urban areas has gained increasing attention^13^. Efforts have been devoted to researching the spatial distribution, ecological risks and human health risks of soil heavy metal pollution in cities^15,23^.

Soil heavy metal pollution studies have mostly used contamination to background value ratios^24,25^, GIS software for spatial distribution estimation^8,16^, driving factors analysis with multivariate statistics^26^, meta-analysis^14^ and ecological health risk analysis^27,28^ to determine pollution levels. Spatial statistics is a powerful tool for estimating the correlations among spatial factors, and some studies have tried to estimate the sources of heavy metal pollutants with multivariate geostatistical analysis methods^29,30^.

The Liaoning central urban agglomeration was one of the first Chinese urban agglomerations. It has an area of 6.5 × 104 km^2^, constituting 44.5% of the total area of Liaoning Province, and includes seven cities: Anshan, Benxi, Fushun, Liaoyang, Shenyang, Tieling and Yingkou. All these cities are heavy industry or mining cities. The Liaoning central urban agglomeration has developed heavy industry since the 1930s due to its abundant local iron, coal and oil resources. The heavy industries in Liaoning Province and all of Northeast China are mainly distributed in this area. In 2017, the population was 2.17 × 10^7^, and the GDP of the Liaoning central urban agglomeration accounted for 50.02% of the total GDP of Liaoning Province. Environmental protection did not receive enough attention before the 1990s; extensive anthropogenic and industrial activities have resulted in many environmental problems, such as water, soil, and air pollution. Many efforts have been made to address water and air pollution in recent years; however, soil pollution control needs more attention in this region. The Hu-Taizi River watershed covers most of the Liaoning central urban agglomeration, except Tieling city.

Few studies have been conducted in urban agglomeration areas, especially within the different land uses of an “urban agglomeration watershed”. Meanwhile, spatial distribution factors were proposed to analyze the potential pollution sources with the CCA method. This study addressed three goals: (1) to evaluate the overall and individual land use pollution conditions of As, Hg, Cd, Cu, Zn, Cr, Pb, and Ni based on the local natural background values in the Hu-Taizi River watershed, (2) to characterize the spatial distribution of these eight heavy metal elements with GIS, and (3) to assess the ecological risk potential sources with multivariate statistical methods.

## Materials and methods

### Study area and sampling sites

**Figure 1:**
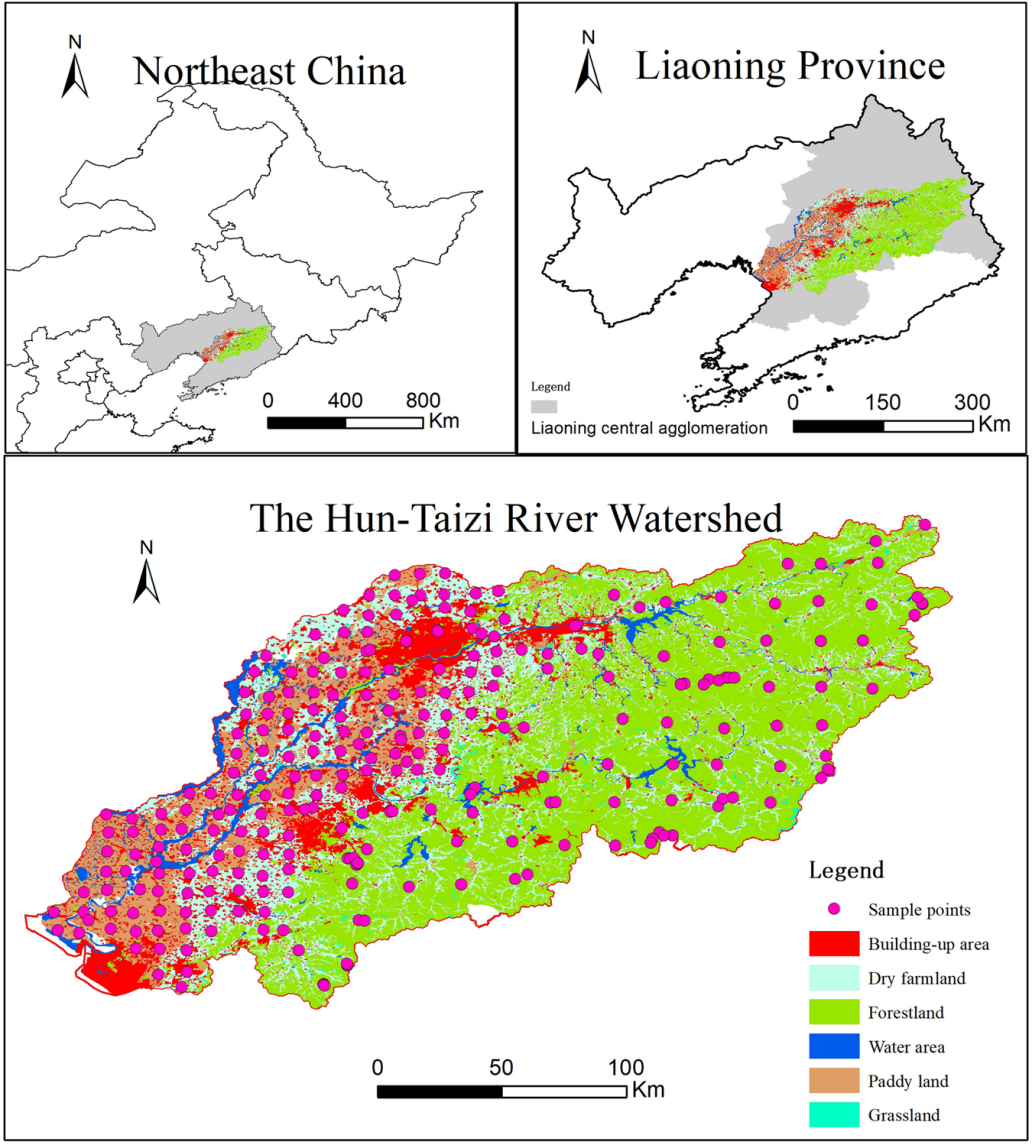
Location of the study area.

The Hun-Taizi River watershed locates in Liaoning Province, Northeast China, and is a sub-basin of the Liao River basin. The watershed area is 2.73 × 10^4^ km^2^, including Anshan, Benxi, Fushun, Liaoyang, Shenyang, and Yingkou cities. The study area includes two main rivers: the Taizi River and the Hun River (Fig. 1). The lengths of the Hun River and the Taizi River are 415 km and 413 km, respectively. The two rivers flow from east to west, following the topography from low hills in the eastern part of the province to the alluvial plain in the central and western parts. The land use and cover are characterized by forestland concentrated in the eastern part, farmland in the central and western parts, and urban areas along the rivers. The sampling points are presented in Fig. 1.

### Sampling and assay

We collected 272 surface (0–5 cm) soil samples considering land use and distribution evenness (Fig. 1). Three duplicate soil samples were collected and mixed at each sampling point. The geographic positions were recorded using portable GPS units. The samples were completely air-dried in a storage room, and any stones and residual roots were removed by hand. Afterward, the samples were sieved with a 1 mm mesh. The samples were kept in sealed brown glass bottles and stored at −4 °C. The samples were analyzed one month after collection.

One standard reference and one reagent blank sample were included in the heavy metal concentration test for data accuracy and precision. The test quality assurance was controlled with the soil standard reference material (GBW07401, GSS-1) obtained from the Center of National Standard Reference Material of China. Soil samples were digested with a microwave digestion instrument (CEM Inc., Matthews, NC, USA) and prepared for the determination of elements. The soil samples Hg and As were measured by HG-AES, while Cr, Cu, Ni, Pb, Zn, and Cd were measured by inductively coupled plasma mass spectrometry (ICP-MS, PerkinElmer, Waltham, MA, USA). The instrument detection limits (mg/kg) of Cu, Zn, Pb, Cd, Ni, Cr, Hg, and As are 0.04, 0.04, 0.1, 0.001, 0.04, 0.04, 0.001, and 0.01, respectively. Each heavy metal solution was tested three times. The data were acceptable under the condition of a relative standard deviation <5%; if the standard deviation did not meet that condition, the soil sample was tested again.

Landsat images (Landsat 7 and 8) from 2016 were used to derive thematic land-use maps. The maps included six land-use categories: paddy, water area, dry farmland, forestland, built-up area and grassland. The interpretation accuracy was 89.7% based on 650 field survey points. The research also used 1:50 000 topographic maps from 2015 to locate mines and roads. The locations of sewage treatment plants and polluting factories were obtained from the Department of Ecology and Environmental Protection of Liaoning Province. A 1:50 000 digital elevation model (DEM) of the study area was obtained from the Liaoning Surveying and Mapping Bureau. Slope and aspect maps were calculated with the DEM using ArcGIS version 9.1 software.

### Soil pollution standard values

There are two national soil environmental quality standards: the soil environmental quality-risk control standard for soil contamination in agricultural land (GB 15618–2018) (Table 1) and the soil environmental quality-risk control standard for soil contamination in development land (GB 36600–2018). This study did not consider land use planning, so the standard for development land was not considered. The GB 15618–2018 standard determines the environmental risk value of agricultural land, which is divided into paddy fields and other agricultural lands. The first natural background values in the study area were obtained in 1986, and the values were obtained out based on soil types (Table 2)^24^. The values in the standards were higher than the natural background values of soils for estimating the environmental risk^31^. The soil samples were collected from different land use types, not only agricultural land, in the study area; moreover, the national standard was decided based on the soil conditions throughout the whole country. Therefore, the local background values were selected for use in order to estimate the degree of soil pollution and potential ecological risk at the watershed scale.

**Table 1:** Soil environmental quality -Risk control standard for soil contamination in agricultural land (mg/kg) (GB 15618–2018) (6.5 < PH < 7.5).

	As	Cd	Cr	Cu	Hg	Ni	Pb	Zn
Paddy field	25	0.6	300	200	0.6	100	140	250
Other	30	0.3	200	100	2.4	100	120	250

**Table 2:** Local natural background values in different soil types (mg/kg).

	As	Cd	Cr	Cu	Hg	Ni	Pb	Zn
Brown soil	10.590	0.118	51.740	23.740	0.055	28.250	24.220	57.750
Paddy soil	9.070	0.128	66.720	21.650	0.081	29.060	29.440	56.650
Meadow soil	8.390	0.129	68.320	23.390	0.088	28.280	20.970	71.810

### Evaluation of the contamination degree

#### Potential ecological risk index estimation

The RI was estimated based on the method proposed by Hakanson^27^, which has been widely used in many studies^28,31^. The RI was calculated using the following equation:

1 $$RI=\mathop{\sum }\limits_{i}^{m}{E}_{r}^{i}=\mathop{\sum }\limits_{i}^{m}{T}_{r}^{i}\times {C}_{f}^{i}=\mathop{\sum }\limits_{i}^{m}{T}_{r}^{i}\times \frac{{C}^{i}}{{C}_{n}^{i}}$$

where RI is the potential ecological risk index, which is the sum of the individual RIs for the potential ecological risks. The classification threshold value was set based on previous studies^27,32^ (Table 3). ${E}_{r}^{i}$ is the potential ecological risk of pollutant *i*; ${T}_{r}^{i}$ is the toxic response factor of pollutant *i* presented by Hakanson; ${C}_{f}^{i}$ is the pollution index of pollutant *i*; ${C}^{i}$ is the concentration of pollutant *i*; and ${C}_{n}^{i}$ is the evaluation reference value of pollutant *i*, which was the natural background value in the study area.

**Table 3:** Risk factors and potential ecological risk classification.

${E}_{r}^{i}$	RI	Potential ecological risk
<40	<70	low
40–80	70–140	moderate
80–160	140–280	considerable
160–320		high
≥320	≥280	very high

The ${T}_{r}^{i}$ values for As, Cd, Cr, Cu, Hg, Pb, Ni and Zn were 10, 30, 2, 5, 40, 5, 5 and 1, respectively.

### Potential sources

#### Statistical analysis

The heavy metal concentrations were interpolated to spatial maps for ecological risk calculation and effect factor analysis with the kriging method in ArcGIS. The data format of the grid used a cell size of 30 m. Two hundred random sampling points were used as training data for map interpolation. The remaining 72 sampling points were used as test points to estimate the accuracy of the interpolation results. The average concentrations of heavy metals in different land use categories, except in the water area, were analyzed based on the heavy metal pollutant interpolation maps and the land use map with ArcGIS.

Pearson’s correlation analysis and principal component analysis (PCA) were used to divide the heavy metals into different groups. The results of the PCA were diagnosed with the Kaiser-Meyer-Olkin (KMO) procedure and Bartlett’s test. Canonical correspondence analysis (CCA) was performed using CANOCO 4.5^33^ to study the interactions between the eight heavy metals and the effect factors. The data were statistically analyzed using the R platform.

### Spatial distribution effect factors

We tried to estimate the potential sources of pollutants through spatial effect factors. Many factors influence the spatial distribution and migration of heavy metal elements in surface soils. The Hun-Taizi River watershed is an urban agglomeration region. A total of 10 spatial effect factors from three aspects were chosen for analysis. Two factors were chosen to describe urbanization, including distance to city and distance to villages. Four factors were chosen to represent geographic conditions, including slope, DEM, distance to the river and distance to reservoirs. Four factors were chosen to represent anthropogenic activities, including distance to main polluting factories, distance to sewage treatment plants, distance to mines and distance to roads. The effect factor maps were analyzed using a grid data format with a cell size of 30 m. A total of 1000 random points were generated within the boundary of the study area, which was used to extract the values of the RI and effect factors with ArcGIS.

## Results and Discussion

### Heavy metal concentrations

The results showed that the mean concentrations of heavy metals were as follows, in declining order: Zn (79.85 ± 32.33) > Cr (65.05 ± 25.23) > Cu (40.97 ± 22.54) > Pb (30.18 ± 13.51) > Ni (29.16 ± 12.33) > As (7.21 ± 3.05) > Cd (0.23 ± 0.25) > Hg (0.12 ± 0.09) (Table 4). Based on the national standard (GB 15618–2018) (Table 1), only the concentration of Cd was over the standard value. Cd was over the standard value in 19.12% of the 272 total sampling points, and the points with excess Cd were located in the suburbs of Shenyang city^31^. However, the proportion of points in which As, Cd, Cr, Cu, Hg, Ni, Pb and Zn were over the local soil background values (Table 2) were 24.54%, 71.43%, 63.37%, 85.71, 70.33%, 53.11%, and 72.16%, respectively. Table 4 shows that the concentrations of Zn, Cr, Cu, Pb and Ni varied greatly and had large standard deviations. A total of 125, 61 and 86 sampling points located in brown soil, paddy soil and meadow soil, respectively. Compared with the local environmental background values for the corresponding soil categories (Table 2), the mean concentrations of As, Cd, Cr, Cu, Hg, Pb, Ni and Zn were 0.68-, 1.98-, 1.26-, 1.73-, 2.10-, 1.03-, 1.25- and 1.38-fold higher, respectively (Table 4). The average concentration of all pollutants was higher than the local natural background values, except that of As. The average concentrations of the pollutants declined in the order Hg > Cd > Cu > Zn > Cr > Pb > Ni. All the maximal values of the eight pollutants were higher than the local natural background values, and the ratios occurred in the following decreasing order: Cd > Hg > Cu > Pb > Cr > Zn > Ni > As, which were 16.61-, 12.18-, 5.37-, 3.75-, 3.86-, 3.18-, 3.15- and 1.89-fold higher, respectively, than the local background values. The percentages over the local natural background values in the 272 sampling points were, in decreasing order: Cu (70.96%) > Zn (58.46%) > Pb (58.09%) > Cd (56.99%) > Hg (56.25%) > Cr (48.90%) > Ni (38.60%) > As (13.24%). The results showed that Hg and Cd had high concentrations at many sampling points. Meanwhile, the Cu, Zn, Pb, Cd and Hg values in over half of the samples in which they occurred were higher than the local natural background values.

**Table 4:** Statistical description of heavy metal pollutants in the study area.

Heavy metal	Concentrations(mg/kg)				SD	Ratio of mean to LNBV	Ratio of Max to LNBV	Percent of over LNBV
	Min	Max	Median	Mean				
As	1.48	20.00	6.73	7.21	3.05	0.68	1.89	13.24
Cd	0.02	1.96	0.14	0.23	0.25	1.98	16.61	56.99
Cr	9.21	164.60	61.97	65.05	25.23	1.26	3.18	48.90
Cu	3.16	127.50	34.73	40.97	22.54	1.73	5.37	70.96
Hg	0.01	0.67	0.09	0.12	0.09	2.10	12.18	56.25
Ni	5.32	89.00	27.20	29.16	12.33	1.03	3.15	38.60
Pb	0.32	93.58	27.95	30.18	13.51	1.25	3.86	58.09
Zn	5.61	215.62	76.19	79.85	32.33	1.38	3.73	58.46

SD: standard deviation; LNBV: local natural background values.

### Comparisons with other basins

Some surveys were carried out in basin boundary, such as the upper Yangtze basin, the Wei River basin in the western part of the Yellow River basin and the Raohe basin in the lower Yangtze basin (Table 5). Comparing the heavy metal concentrations in the study area to those in the other three basins showed that the average concentration of Cd was at the high pollution level, but Cr was at the low pollution level. As, Cu, Hg, Ni, Pb and Zn had similar average concentrations. The maximal values of Cd, Cr, Cu, Ni and Zn placed them at the high pollution level. The comparison showed that Cd was the primary pollutant and that Cd, Cr, Cu, Ni and Zn had high pollution level points in the study area.

**Table 5:** Concentrations of heavy metals in the upper Yangtze, Raohe and Wei River basins (mg/kg).

		As	Cd	Cr	Cu	Hg	Ni	Pb	Zn
Upper Yangtze Basin^7^	Mean	6.21 ± 3.21	0.33 ± 0.10	75.49 ± 12.03	26.99 ± 8.59	0.08 ± 0.002	35.24 ± 9.18	27.90 ± 3.00	87.91 ± 15.77
	Max.	32.77	1.57	144.40	106.50	1.79	96.39	59.30	238.50
Raohe Basin^9^	Mean	78.52	0.51	35.26	197.21	—	31.03	39.63	32.31
	Max.	318.05	1.60	97.09	793.52	—	66.35	222.19	72.09
Wei River Basin^38^	Mean	3.89 ± 0.99	0.18 ± 0.24	50.12 ± 7.58	26.89 ± 6.93	0.07 ± 0.11	12.01 ± 3.69	—	62.33 ± 16.26
	Max.	5.86	1.20	73.40	35.60	0.39	19.08	—	93.30

Different boundaries have been used to define study areas, such as watersheds, agricultural soils, mining areas, industrial areas, river deltas, ocean bays, urban areas, and suburban areas^3,34^. Which boundary is the most suitable for soil heavy metal estimation? Hydrological processes and soil erosion are the main migration carriers of heavy metals and are usually studied at the watershed or basin scale. Moreover, most cities and urban agglomerations are located along rivers^1^. Therefore, the watershed boundary is the most suitable boundary for soil pollution estimation, source analysis and eco-environmental comprehension.

### Spatial distribution of heavy metals and ecological risk

**Figure 2:**
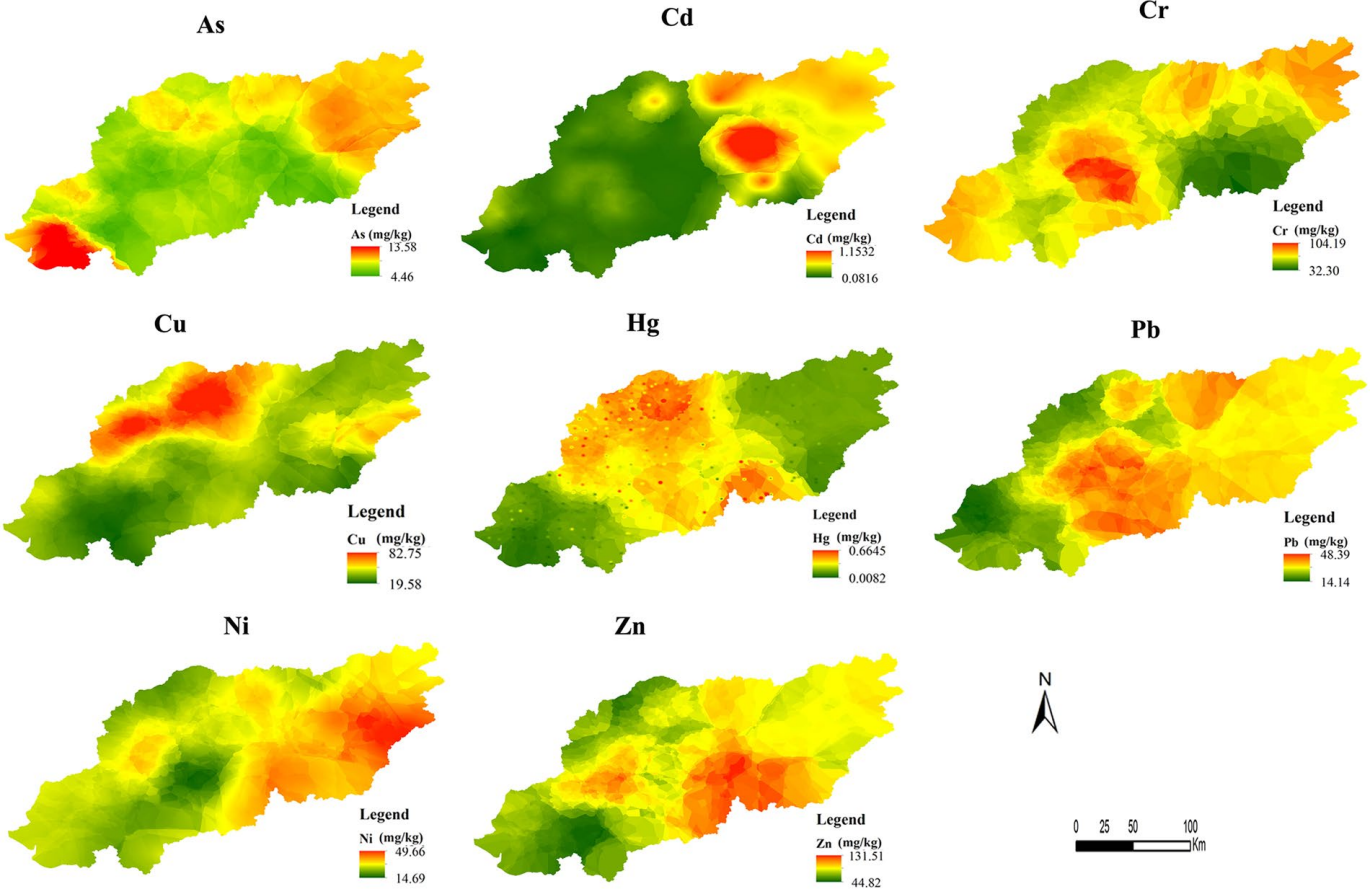
Spatial distribution of eight heavy metals in the study area.

**Figure 3:**
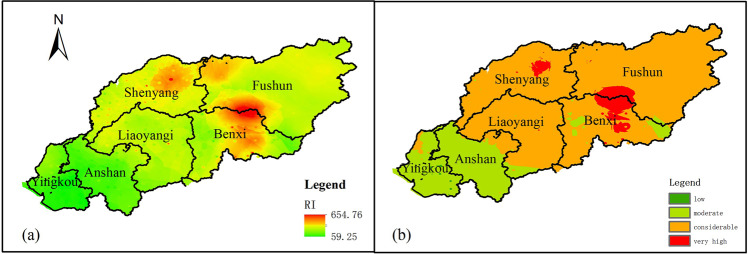
Spatial distribution of the potential ecological risk index (**a**) and classification (**b**).

The spatial distribution maps were generated with 200 random sampling points using the kriging method. The maps were shown in Fig. 2. The map accuracies for As, Cd, Cr, Cu, Hg, Pb, Ni and Zn based on the other 72 sampling points were 83.46%, 88.32%, 79.56%, 84.86%, 78.13%, 82.51%, 80.18% and 83.55%, respectively, which showed that the results were acceptable. A potential ecological risk index map (Fig. 3(a)) was estimated based on the RI calculation equation, and the ecological risk index was reclassified into four potential ecological risk levels (Fig. 3(b)). As shown in Fig. 3, two high-value regions were located near Shenyang city and the junction area between Benxi and Fushun, which accounted for 4.33% of the study area. The percentages of the study area that were considered to have low, moderate and considerable potential ecological risk were 0.11%, 20.84% and 74.73%, respectively. Cd was the main pollutant due to its mean concentration and potential ecological risk contribution (${T}_{r}^{i}$ = 30). As shown in Fig. 2 (Cd) and Fig. 3, the spatial distributions of Cd and the RI were similar, meaning that the RI was mainly influenced by Cd in the study area.

### Heavy metal concentrations in different land use categories

Based on the results of the interpretation of Landsat images, the areas of paddy, water area, dry farmland, forestland, built-up area and grassland were 3864.54 km^2^ (14.12%), 1171.68 km^2^ (4.11%), 6422.53 km^2^ (22.51%), 13108.39 km^2^ (47.91%), 3654.22 (12.81%) km^2^, and 312.77 km^2^ (1.10%), respectively. Forestland occupied almost half of the study area and was mainly distributed in the eastern and southern mountainous areas (Fig. 1). Farmland, including dry farmland and paddies, accounted for 36.05% of the study area and located in the central and west alluvial plain areas. The built-up area accounted for 12.81% of the total area because the study area covers most of the Central Liaoning Urban Agglomeration, which is one of the Chinese urban agglomerations.

The average concentrations of all the heavy metal pollutants, except Ni, were obviously higher in human-dominated land use categories, including built-up area, paddy and dry farmland (Table 6). The results showed that the spatial distributions of As, Cd, Cr, Cu, Hg, Pb, and Zn were significantly affected by anthropic activities. The high concentration area of Ni located in the southeastern part of the study area, which is mainly covered by forests. Forestland had the lowest concentration values for all heavy metal pollutants except Ni. Grassland had similar heavy metal concentration levels as forestland. The highest mean concentrations of Cu, Hg and Zn were distributed in the built-up area. Paddy areas had the highest mean concentrations of Cr and Pb.

**Table 6:** Mean concentrations of heavy metal pollutants in different land use categories.

	As	Cd	Cr	Cu	Hg	Ni	Pb	Zn
Forestland	6.98	0.18	62.64	36.63	0.10	33.19	26.16	77.10
Grassland	7.20	0.21	64.25	37.00	0.10	28.55	28.56	77.39
Built-up area	7.52	0.21	67.73	45.33	0.12	28.25	30.35	89.95
Paddy	7.62	0.24	69.10	43.07	0.11	28.49	34.54	78.20
Dry farmland	7.43	0.35	64.27	41.14	0.12	29.13	31.29	79.37

### Potential sources of heavy metals

**Figure 4:**
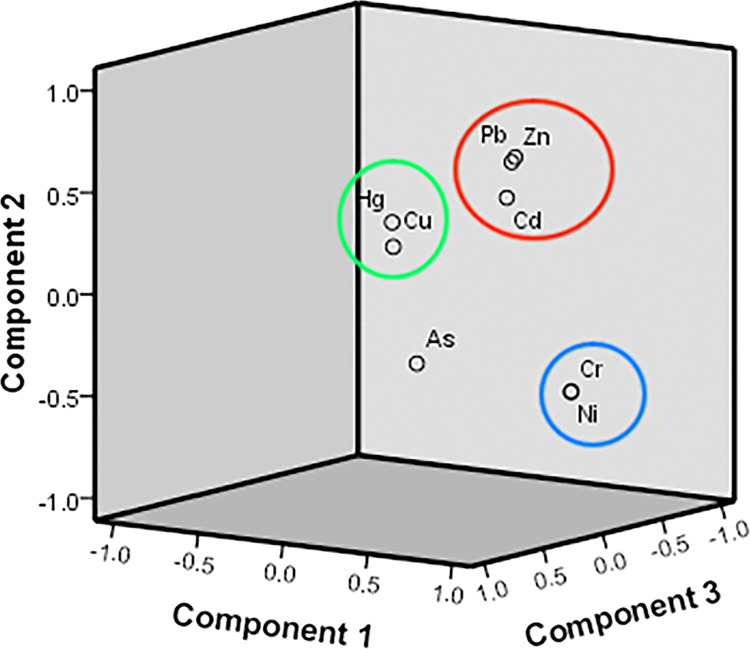
Loadings of the first components obtained from PCA.

The PCA method and Pearson’s correlation matrix were used to identify similar groups of soil heavy metals, and the results are shown in Fig. 4 and Table 7. These nonparametric tests were analyzed for nonnormally distributed data using the Shapiro-Wilks test. The significant correlations were estimated with Pearson’s correlation analysis (p < 0.05): Hg and Cu (r = 0.367), Ni and Cr (r = 0.999), Pb and Cd (r = 0.253), Pb and Hg (r = 0.178), Zn and As (r = −0.163), Zn and Cd (r = 0.236), Zn and Cu (r = 0.236), and Zn and Pb (r = 407) at p < 0.01, as well as Pb and As (r = −0.143) and Zn and Hg (r = 0.132). Pollutants with significant correlations may come from similar or even the same pollution sources.

**Table 7:** Correlation coefficients between heavy metal pollutants (N = 272).

	As	Cd	Cr	Cu	Hg	Ni	Pb	Zn
As	1							
Cd	0.058	1						
Cr	0.082	0.027	1					
Cu	0.029	0.060	0.089	1				
Hg	−0.117	0.028	0.057	0.367^**^	1			
Ni	0.082	0.036	0.999^**^	0.093	0.061	1		
Pb	−0.143^*^	0.253^**^	0.059	0.044	0.178^**^	0.056	1	
Zn	−0.163^**^	0.236^**^	0.086	0.183^**^	0.132^*^	0.094	0.407^**^	1

Levels of significance: *p < 0.05. **p < 0.01.

The results of the PCA were valid, as indicated by KMO = 0.792 and Bartlett’s test < 0.001. The results of the PCA showed that the first principal component (PC1), second principal component (PC2) and third principal component (PC3) explained 76.34% of the total variance. PC1 was correlated with Cr and Ni and explained 26.60% of the total variance. PC2 was correlated with Pb, Zn and Cd and explained 21.50% of the total variance. PC3 was correlated with Hg and Cu and explained 15.09% of the total variance. According to the PCA results (Fig. 4) and Pearson’s correlation analysis (Table 6), three groups were identified: Hg and Cu; Pb, Zn and Cd; and Ni and Cr. The pollutants in the same group shared similar potential sources, mainly anthropogenic activities, especially industrial production.

**Figure 5:**
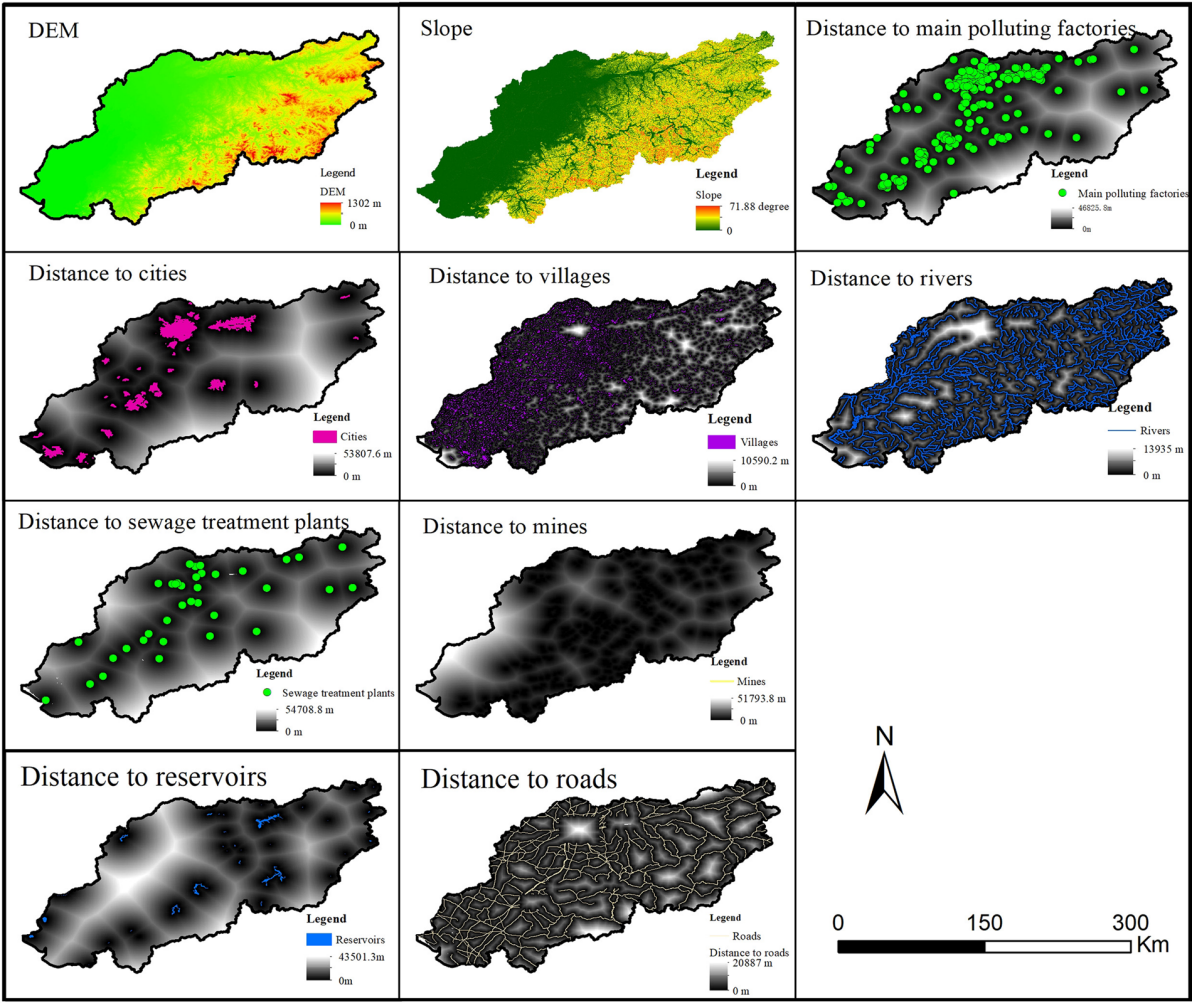
Maps of effect factors.

**Figure 6:**
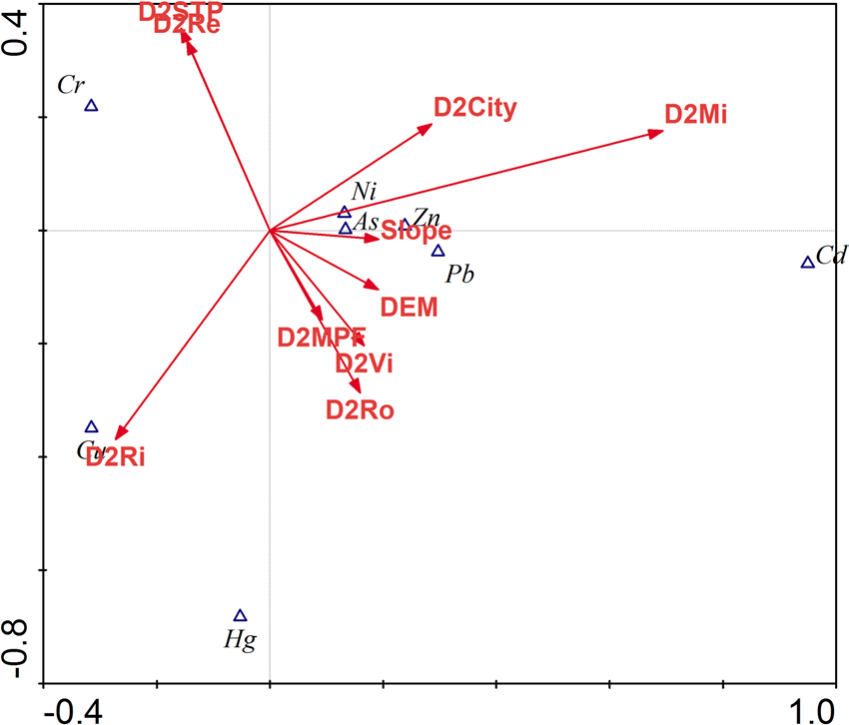
Canonical correspondence analysis between eight heavy metal concentrations and effect factors. Abbreviations: D2STP – Distance to sewage treatment plants, D2Re – Distance to reservoirs, D2City – Distance to cities, D2Mi – Distance to mines, D2MPF – Distance to main pollution factories, D2Vi – Distance to villages, D2Ro – Distance to roads, D2Ri – Distance to rivers.

Ten environmental variables (Fig. 5) were chosen to analyze the effect factors and identify the sources of the eight heavy metals with CCA. The CCA presented an evident and graphic representation of the correlations between pollution contaminants and environmental effect factors^26^. As shown in Figs. 4 and 6, heavy metals were mainly divided into three groups according to the results of the PCA. The results showed roughly three directions for these environmental variables. Distance to mines was the strongest factor influencing the spatial distribution of As, Cd, Ni, Pb, and Zn. The high-value area for Cd and the RI was the area adjacent to Fushun and Bexin cities, which is the main iron mining area.

The spatial distribution of Cu was strongly influenced by the environmental factor of distance to the river. The pollution area for Cu was probably caused by factory emissions and the livestock industry. As Fig. 2 (Cu) shows, the high concentration area for Cu was along the Hun River. The cities of Fushun and Shenyang along the Hun River have been heavy industrial cities for the past several decades and are also the location of intensive livestock and poultry breeding activity. The average Cr concentration in the study area was only 1.26 times the local background value; meanwhile, no high pollution areas located in the study area. The spatial distribution of Pb was affected by slope, DEM, roads, villages, and the distribution of the main polluting factories, which means that multiple sources and roads were the most important factors influencing the distribution of Pb.

The heavy metals concentration in surface sediments (0–15 cm) were carried out in Liaohe River watershed, which contains Hun-Taizi River watershed^35,36^. The average background levels of Cr, Cu, Ni, Pb, and Zn were 32.6, 11.1, 13.1, 16.3, and 37.8 mg/kg, which were all lower than the values in 1986. The Liaoning central urban agglomeration is one of the historical Chinese industrial regions where industrialization began in the 1930s due to the coal, iron, and magnesium ore mines distributed in this area^37^. Industrialization developed over 80 years in the study area, and surface soil accumulation can only be used to estimate the present heavy metal pollution situation. More attention should be focused on the trajectory of historical soil pollution processes in future studies. For this purpose, future work should focus on undisturbed river or lake sediments for soil profile analysis.

## Conclusions

Based on these sampling sites and the national standard GB 15618–2018, there was only a contamination area for Cd in the study area. The average concentrations of heavy metals were higher than the local natural background values, except the average As concentration. Two high-value RI regions accounted for 4.33% of the study area and were mainly determined by the Cd distribution. Cd was the main pollutant in the study area due to its high concentration area and potential ecological risk contribution. Compared with surveys in other basins, Cd was present in higher concentrations. The percentages of the study area considered to be at low, moderate and considerable potential ecological risk were 0.11%, 20.84% and 74.73%, respectively. The pollutants were clustered into three groups based on correlation analyses and possible sources: Hg and Cu; Pb, Zn and Cd; and Ni and Cr. Based on spatial analysis and the CCA method, the mine distribution was the strongest effect factor influencing the spatial distributions of As, Cd, Ni, Pb and Zn. The spatial distribution of Cu was strongly influenced by the distance to the river. Watersheds are the best study boundaries for surveying and understanding the distribution processes of heavy metals. The trajectory of the historic soil pollution mechanisms in the study area should be studied and identified in the future. The methods in this study are useful for the estimation of potential sources of soil heavy metal pollutants with spatial effect factors using GIS and statistical methods.

## Data Availability

The datasets analyzed during the current study are available from the corresponding author on reasonable request.
